# Utilization of community pharmacy space to enhance privacy: a qualitative study

**DOI:** 10.1111/hex.12401

**Published:** 2015-08-31

**Authors:** H Laetitia Hattingh, Lynne Emmerton, Pascale Ng Cheong Tin, Catherine Green

**Affiliations:** ^1^ School of PharmacyFaculty of Health SciencesCurtin UniversityPerthWAAustralia

**Keywords:** community pharmacy, consultation area, pharmacy layout, privacy, qualitative, space

## Abstract

**Background:**

Community pharmacists require access to consumers’ information about their medicines and health‐related conditions to make informed decisions regarding treatment options. Open communication between consumers and pharmacists is ideal although consumers are only likely to disclose relevant information if they feel that their privacy requirements are being acknowledged and adhered to.

**Objective:**

This study sets out to explore community pharmacy privacy practices, experiences and expectations and the utilization of available space to achieve privacy.

**Methods:**

Qualitative methods were used, comprising a series of face‐to‐face interviews with 25 pharmacists and 55 pharmacy customers in Perth, Western Australia, between June and August 2013.

**Results:**

The use of private consultation areas for certain services and sensitive discussions was supported by pharmacists and consumers although there was recognition that workflow processes in some pharmacies may need to change to maximize the use of private areas. Pharmacy staff adopted various strategies to overcome privacy obstacles such as taking consumers to a quieter part of the pharmacy, avoiding exposure of sensitive items through packaging, lowering of voices, interacting during pharmacy quiet times and telephoning consumers. Pharmacy staff and consumers regularly had to apply judgement to achieve the required level of privacy.

**Discussion:**

Management of privacy can be challenging in the community pharmacy environment, and on‐going work in this area is important. As community pharmacy practice is increasingly becoming more involved in advanced medication and disease state management services with unique privacy requirements, pharmacies’ layouts and systems to address privacy challenges require a proactive approach.

## Introduction

Australia has approximately 5450 community pharmacies that provide a range of medication‐related services such as dispensing of prescriptions, counselling about appropriate use of medicines, provision of healthcare information and supply of non‐prescription medicines.^1^ The Commonwealth Government restricts the location of community pharmacies through *Pharmacy Location Rules* to facilitate equitable access to medication services regardless of consumers’ location.^2^ Furthermore, requirements regarding pharmacy premises are specified under state and territory legislation.^3^ In most jurisdictions, pharmacies require an area for private consultation. For example, in Western Australia, Section 7 of the Pharmacy Regulations 2010 (WA) specifies as follows:

The premises are to have an area in which a consultation conducted by a pharmacist is not reasonably likely to be overheard by a person not a party to the consultation.

The need to regulate community pharmacy ‘space’ to ensure privacy is justified by pharmacists’ use of information about consumers’ medicines and health conditions to make informed decisions regarding treatment options. Open communication between consumers and pharmacists is ideal, although consumers and carers are only likely to disclose relevant information if they feel that their privacy requirements are being acknowledged and adhered to.^4^ Strict adherence to Australian community pharmacy privacy requirements is somewhat complicated by the retail element as pharmacists and pharmacy staff provide services mostly in the public eye.

Changes in the healthcare landscape over recent years have resulted in the expansion of the role of community pharmacists. Certain new services have premises requirements, for example government‐funded in‐pharmacy medication review services introduced in 2012 require a screened area or separate room that is distinct from the general public area of a pharmacy.^5^ Recent changes to legislation that enable pharmacist‐administered influenza vaccination services in Western Australia similarly require pharmacies to have a screened area or private room with sufficient space to administer vaccinations.^6^

In addition to the physical pharmacy space requirements, pharmacy staff must comply with privacy processes and procedures as specified in the Privacy Act 1988 (Cth). The Act defines personal information as information or an opinion about an individual who is identified or is ‘reasonably identifiable’, and includes a person's name, address, Medicare number or any other health information such as notes or opinions.^7^ Pharmacists must also comply with the *Code of Conduct for Registered Health Practitioners*, which states health professionals must ‘protect the privacy and right to confidentiality of patients and clients’.^8^ The Pharmaceutical Society of Australia's *Code of Ethics and Professional Practice Standards* also require safeguarding the consumer's right to privacy and confidentiality at all times.^9^, ^10^

### Pharmacy privacy research

A 2004 systematic review of community pharmacy services highlighted consumers’ concerns about the level of privacy and that utilization of community pharmacies might depend on a pharmacy's facilities for a private discussion.^11^ The concept of privacy in pharmacies has been the subject of a number of studies, mostly in the context of a particular product, service or medical condition. A recent Australian study involving 74 mental health consumers and carers found consumers are likely to form trusting relationships with community pharmacy staff if they perceive pharmacies as safe health spaces.^12^ The research identified a need for pharmacy staff to be more discreet when calling out patient names, having private conversations in the pharmacy and exposing medication packs being purchased or issued.^13^ Participants of a 2010 United Kingdom (UK) community pharmacy‐based cardiovascular screening study similarly identified concerns about confidentiality and lack of privacy as barriers to participating in screening services.^14^ A 2001 pharmacy‐led intervention in The Netherlands focused on pharmacies as sources of information, reporting lack of pharmacy privacy led to reluctance to ask questions.^15^

A recent Australian study involving consumer focus groups identified a lack of privacy as a major logistical barrier to consumers’ participation in chronic disease management programmes.^16^ Similarly, in the UK, research to enhance the utilization of community pharmacy services identified lack of privacy and confidentiality as crucial obstacles that could inhibit service utilization.^17^

A number of studies have focused on pharmacy privacy and the provision of emergency hormonal contraceptives (the ‘morning‐after pill’). A ‘mystery shopper’ Australian study reported low use of private and semi‐private consultation areas.^18^ Other emergency hormonal contraceptive studies similarly highlighted concerns around lack of pharmacy privacy.^19^, ^20^, ^21^, ^22^, ^23^

These studies indicate that, from the consumer perspective, there is a need for increased sensitivity about privacy requirements in community pharmacy practice. Although some of the newer professional services require the use of separate consultation areas, it is unknown whether these areas are being used as intended. UK research found consultation rooms were perceived as less accessible than originally envisioned and were being used for other purposes or were not patient‐friendly, making their utilization challenging.^24^

Although there are regulatory requirements about layout and procedures to protect consumers’ privacy in pharmacies, the literature suggests compliance with privacy in pharmacy practice is challenging.^12^, ^13^, ^14^, ^15^ A need was therefore identified to explore community pharmacy privacy practices, experiences and expectations from the perspectives of both pharmacists and pharmacy consumers.

## Methods

This study utilized qualitative methods comprising a series of interviews with pharmacists and pharmacy customers in Perth, Western Australia, to explore privacy practices, experiences and expectations and the utilization of available space to achieve privacy. Qualitative methodology allowed exploration of participants’ views. Low‐risk ethical approval was granted (PH‐17‐13).

### Participant recruitment

The selection of pharmacies followed a purposive sampling approach to cover a range of characteristics and population demographics. In 2013, there were 584 pharmacies in Western Australia (WA), 424 of which were in the capital city, Perth.^25^ Perth metropolitan pharmacies were categorized geographically as north‐west, south‐west, north‐east, south‐east and central. A further categorization process followed whereby pharmacies were allocated according to socio‐economic area (based on median house price for the suburb), size of pharmacy (based on average prescription number and number of staff), location (major/smaller shopping centre or street front) and whether pharmacies were independently owned or member of a brand or banner group. Pharmacies from each category were selected and a shortlist of 66 pharmacies created. Pharmacies were limited to approximately 25 km from the city centre for logistical reasons.

Pharmacist managers were initially approached by telephone and if interested to participate were emailed copies of the participant information sheet and consent form. Face‐to‐face interviews were conducted during business hours at the pharmacies at times convenient to the pharmacists, and participants received a $50 gift card in recognition of their involvement.

After each pharmacist's interview, the pharmacist and researcher approached pharmacy consumers to be interviewed. If the consumers agreed to the interview, they were provided with an information sheet and interviewed by the researcher (CG) during the same visit. Interviews took place face‐to‐face in the pharmacy in an area comfortable to the consumer, either in a quiet area, a semi‐private area or a consulting room if available. All participants signed a consent form before being interviewed. Consumer data were not shared with pharmacy staff.

The recruitment of pharmacist and consumer participants ceased when data saturation was perceived by the research team; qualitative methods suggest this is achieved with samples of 20–25 participants.^26^

### Interview guides

A semi‐structured guide with 12 open‐ended questions with prompts for elaboration was used for the pharmacist interviews. While qualitative methods generally advocate unstructured interviews, semi‐structured (guided) discussions that allow participants to follow particular lines of reflection, yet within a framework controlled by the interviewer, are commonly applied.^27^ Discussion points were developed with reference to the literature and explored:

1. Pharmacists’ perspectives of the use of various consultation areas in the pharmacy including the use of space for the provision of newer pharmacy services
2. Advantages and disadvantages of using these spaces
3. Pharmacists’ perceptions of consumers’ needs and expectations relating to privacy and confidentiality
4. Examples of situations during which pharmacy staff were unsure about their privacy and confidentiality obligations.

The consumer interview guide consisted of nine open‐ended questions that followed a structured format. Questions to consumers explored are as follows:

1. Consumers’ perceptions of privacy during consultations in pharmacies
2. Satisfaction with privacy and confidentiality in interactions with pharmacy staff
3. Consumers’ knowledge of privacy in relation to pharmacy.

Interview guides were validated by all four team members as well as two academic colleagues, seeking feedback on questions, format and suggestions for improvement. Minor changes regarding the flow of questions were incorporated, and the guides were reviewed for construct validity^27^ by a pharmacist and consumer.

### Data management and analysis

Pharmacist interviews were audio‐recorded, supplemented with notes and diagrams, then de‐identified and transcribed verbatim. Information from consumer interviews was noted on interview guides during the interviews. The interviews were conducted with the participants’ consent but not recorded to minimize discomfort of the consumers when unprepared for the interview.^28^, ^29^

NVivo^®^ (Version 9.0) (NVivo qualitative data analysis software; QSR International Pty Ltd. Version 9, Doncaster, Victoria, Australia) was used to organize the data. Thematic analysis of the data was informed by the general inductive approach^30^ and comprised multiple stages. The data were coded, and emergent themes were noted with supporting quotations within each theme. The themes were organized under distinctive headings addressing the research objectives. To ensure reliability of the analysis, all authors reviewed and agreed upon the themes. This involved members of the research team reading and re‐reading the transcripts to gain an understanding of the broad issues. Specific descriptive topics and themes were developed to capture core messages reported by participants.

## Results

### Participants

Interviews were conducted between June and August 2013. Twenty‐five pharmacists (13 males, 12 females) from 25 community pharmacies participated: 12 were pharmacy managers, 10 were owners and three the pharmacist‐in‐charge. Fourteen had <10 years’ experience, eight had 10–20 years’ experience, and three had more than 20 years’ experience. Thirteen of the participating pharmacies were independently owned; the remaining 12 were members of banner groups. The majority of the pharmacies (*n* = 15) were located as street‐front pharmacies, with seven in shopping centres and three next to a medical centre. The duration of the pharmacist interviews averaged 30 min (17.0–58.5 min).

The 55 consumer participants (on average two from each pharmacy) centred around 55 years of age and comprised 34 females and 21 males. The majority of the participants were regular clients at the pharmacy where they were interviewed. Although most of the consumers visited the pharmacies to obtain prescription medicines 20 of the consumers presented for another reason, that is to purchase a non‐prescription product or ask for advice.

### Themes common to pharmacists and consumers

A number of themes were common to the pharmacists’ and consumers’ responses:

1. Support for the use of allocated private consultation areas
2. Challenges with overhearing conversations
3. Visibility of people and products
4. Judgement regarding required privacy.

These themes are presented with illustrative quotations: pharmacists’ responses are represented with ‘P’ and consumers’ with ‘C’.

### Support for the use of allocated private consultation areas

The majority of the pharmacies had allocated spaces for private discussions, ranging from a seated semi‐private consultation area (in a quiet corner of the pharmacy) to a semi‐private booth (partitioned area at counter) or a separate consultation room (Table 1). Three of the pharmacies had more than one area whereas four of the pharmacies had no specific allocated space for private discussions.

**Table 1:** Private consultation areas of participating pharmacies

Space	*n* a
Consultation room	10
Seated consultation area in pharmacy	6
Consultation room as well as seated consultation area	3
Booth	2
No dedicated private area	4

a *n* = 25 pharmacies.

#### Pharmacists’ comments

Pharmacist participants reported that the private consultation rooms were used to provide in‐pharmacy medication review services. Private consultation rooms were also increasingly used for discussions sensitive in nature such as the supply of medicines for treatment of vaginal candidiasis and genital herpes, and requests for emergency hormonal contraceptives. Overall, there was agreement between the pharmacists that dedicated space for private or semi‐private discussions was a necessity for the pharmacy profession to progress and embrace expanding roles. Although most pharmacists were positive about private rooms, there were some reservations based on cost (sacrificing high‐rent‐value retail space) and perceived uneasiness of consumers in being taken into a room (Table 2).

**Table 2:** Positive and negative aspects of private consultation rooms identified by pharmacists

Positive aspects	
Support the changing role of pharmacists	…It's [consultations] got to be done in private, that's sort of the changes that I think I'm going to need to move forward. You're going to be a pharmacist sitting out here, having consultations……So, refit… P07 …Well, it's good that we're doing that; it's becoming more of a pharmacist's role in society, but you definitely need a private area, consultation area …… somewhere where you can sit and discuss. That was the purpose for having this room …..Vaccinations ……. I think that is something that we'd be able to do in the pharmacy, we'd be able to roll it out here because of the set‐up that we've got… P11
Enable introduction of new services	…So, we believe that the consulting room will actually make a huge difference in the way of how comfortable we are … and we are actually thinking that we can do some weight management, where we can check the patient's weight and waist measurement… P14
Better‐informed about consumers	…Recently, with talking with mental health customers, I'm now more aware and …. now sitting down and talking to people with mental health, it just makes me understand more. I was not really judging them before, but all the things now fall in place when you sit down and talk to them about other things … and it makes us a better‐informed health professional and a bit more mindful talking to customers… P01
Negative aspects	
Additional workload	…It's not a physical area that's the biggest barrier, it's the workload put on the pharmacists … there has to be enough money in the pharmacy to allow for two pharmacists,essentially. I think that's the only way… P16
Financial barriers	…but then you come back to the cost basis – how do you do this? What do you do? How many people can you afford to employ? Who's going to pay for it? You're paying for a shop per square metre, it's the same everywhere in commercial realty… P21
Consumers feeling uncomfortable	…I think the odd person thinks ‘oh, why do I have to go in there?’ They might feel a bit uncomfortable about it, thinking that we might maybe drawing more attention to them… P11

One pharmacist proposed a solution to overcoming the barrier of having the pharmacist segregated in a consultation room and unable to supervise other activities:

…I have thought that maybe having these [Perspex barriers] up a little bit higher would be good, because people can walk past and see what's happening. But I need to be in here and be able to see out there [into the pharmacy]. It's not the most private, but it's used a lot – I feel it gets good use. It's practical…

P24

The management of consumers who received medicines through staged‐supply services (daily or weekly supply) or who were using opioid substitution therapies varied between pharmacies; these consumers were served at the main or dispensary counters, or in a dedicated semi‐private area.

#### Consumers’ comments

A number of consumers were aware of a private consultation room within the pharmacy as a recent and positive addition:

…When I saw they built this room, I thought it was a good idea, because sometimes people want to be private…

C24

The availability of a private room encouraged some consumers to have more discussions in the pharmacy:

…I wouldn't discuss things out there that I would be bothered with … [but would rather use a private room], not necessarily for confidential things, you just want them to be private…

C19

Consumers who used pharmacies without a private consultation area highlighted a need for such an area within the pharmacy for interactions that may require privacy:

…The only thing I would say is that if it was something of a more serious nature or more personal nature, maybe there's an area of the pharmacy that could be looked at for speaking to somebody who's particularly sensitive or has a serious health issue that they don't want everybody else to know or overhear…

C10

### Challenges with overhearing conversations

Both pharmacists and consumers highlighted pharmacy layout shortcomings that pose difficulties with keeping conversations private.

#### Pharmacists’ comments

Table 3 is a summary of situations described by pharmacists when consumers who were present in the pharmacy could overhear conversations. The majority of pharmacists indicated that counselling was provided at the counter unless a need for greater privacy was identified. However, pharmacy counters were identified as a specific problematic area to be managed to prevent consumers overhearing private conversations:

**Table 3:** Challenging situations for overhearing conversations identified by pharmacists

Small pharmacies	…this particular pharmacy is small, so it's easy for a customer to overhear a conversation from another customer… P10
Consumers with hearing problems	…We had a lady and she couldn't understand…and I was trying to explain to her…and there was a customer waiting behind, and while I was trying to be really quiet, there was only so much [I could lower my voice] or the [lady] couldn't hear … but, as much as you try to keep that private, you can't … you're trying to explain to someone and another person is standing really close and the lady's talking loudly, so, I guess in that way, as much as you try to maintain privacy, you can't… P03
Comments by pharmacy assistants	…My friend, she had a urinary tract infection … and this was at the cashier, in front of everyone, and the [pharmacy assistant] said ‘Can I ask you a personal question – do you go to the toilet after you have sex?’ and my friend … and her husband was, like, ‘I'm out of here.’ And she said she was so embarrassed… P03
Telephone conversations	…if you're on the phone with someone who is hearing impaired and they say ‘speak up, speak up’ then you just don't know who might be listening, and you have to speak up because they can't hear you – and you may get a customer saying ‘well, what was that all about?’ P23 …Because of the high volume of methadone clients, we're constantly communicating [by telephone] with doctors and nurses, prison officers, police … regarding this specific group of people who need a lot of attention… so phone is difficult… P08

…people queuing to pay at the counter and you've got counselling happening at the same time. It's quite difficult in that way; you just have to try to be discreet…

P19

One pharmacist commented on asking consumers to visit the pharmacy during quiet times to discuss specific issues:

… I'll call them to come to the quieter area at a quieter time, like early in the morning or after 5.30 pm – it's a very quiet pharmacy at that time. So that's what I do if they really want to discuss the medication or a serious issue. Because all the customers here, we've known about 10–15 years, so they listen and come after 5.30…

P20

Another challenging situation was to verify the identity of a person requesting information over the telephone:

…How do we know that that's the person they say they are? What kind of information do you get from them?

P03

#### Consumers’ comments

Most consumers indicated they felt comfortable within the pharmacy environment and that their privacy was protected during interactions with the staff. A number perceived this was due to the absence or scarcity of other consumers in the pharmacy at the time they received counselling. However, should the pharmacy have been busy at the time, some reported they would choose a less busy time to ask questions:

…There was no‐one else around. I think if I was not comfortable, I would have left, the pharmacist knows that…

C04

Consumers highlighted staff strategies to enhance protection of their privacy during interactions to make them feel comfortable. These actions included lowering their tone of voice and moving aside to a private area to provide more privacy:

…The centre of the counter seems very full‐on, and there is a lot of activity happening, so whoever we were talking to would always, very discreetly, not imposingly, but very discreetly pull us aside to talk about things…

C01

There were comments from some consumers about having overheard private conversations at the front counter:

…I must admit, I've heard some interesting things along the way…

C32

…I was in a pharmacy in [suburb] once … buying something at the counter, and the pharmacy assistant called out across the shop, which was bigger than this one, ‘Oh, Mr so and so, your methadone's here!’ … that was a bad one…

C43

### Visibility of people and products

Table 4 provides a summary of challenges to overcome information that could be visible to pharmacy customers and some solutions identified by pharmacists. Another issue identified was pharmacy customers recognizing each other. One consumer referred to the challenges of living in smaller towns with only one pharmacy, where pharmacy customers tend to know each other:

**Table 4:** Challenges and solutions to manage visible information identified by pharmacists

Challenges with the space surrounding pharmacy counters, such as consumers being able to observe prescriptions or other products being sold	…Sometimes … I'm talking about blood pressure medication, other people are standing and have a look on the box, so you can't 100% keep it 100% private because you're going to take it out and on the box they can see atenolol is for the blood pressure, so they know this customer has blood pressure… P20
Measures to prevent consumers from seeing other consumers’ dispensed products included asking consumers to sign beforehand and putting the medicines in a bag before handing it to the consumer	…Scripts are treated carefully; like sometimes we try to get the patient to sign it, if they've signed it beforehand, to make sure they're filed away before they're brought out to avoid that. Sort of more discrete medications like DDs [dangerous drugs], dexamphetamines, for example or Viagra^®^ [used for erectile dysfunction], things like that, we put in a bag before it's handed out and then we do have this counselling area for privacy and we've also got the partition section at the end of the counter for privacy as well… P11
Visibility of computer screens was mentioned as an aspect that requires staff awareness, with some of the pharmacists commenting on the use of ‘wait screen’ functions or screen savers in‐between tasks. One pharmacist highlighted the situation when consumers come within close proximity of private information displayed on computer screens	…people walk right to here (entrance to dispensary/consulting room) and I'm like ‘I'm really sorry, but can you just step back?’ because people don't realise that they can see, you know, they can look at the computer screen… P03

…I have not felt comfortable … sometimes you are asked questions that you'd rather not everybody knows… it's just not comfortable especially if you live in a small town. Even if you're buying Panadol^®^, you don't want your neighbour to know – it's none of their business. I don't think they [the staff] consciously talk too loud; it's just if there's too many customers…

C04

### Judgement regarding required privacy

Both pharmacists and consumers recognized the need for sensitivity and professional judgement to facilitate the required level of privacy.

#### Pharmacists’ comments

An increased need for privacy appeared to depend on the nature of the medication being supplied and the pharmacist detecting consumers’ cues. Specific medicines identified that should be managed with extra privacy were the emergency hormonal contraceptive, medicines for erectile dysfunction, medicines for genital herpes simplex infections and dexamphetamine.

Pharmacists generally offered the level of privacy to a consumer that they judged desirable by that consumer, and if this was perceived to be insufficient, responded further:

…You can kind of judge by someone's … body language, whether or not they're comfortable, and, if they're not, you can always take them aside…

P03

…you get a feeling for if the patient needs [more privacy], or isn't comfortable talking about the condition or the medication in front of other people that are standing there, then they're always moved to a separate place or a quiet place and then we go through it…

P06

Consumer variability was highlighted, and pharmacists should use judgement to determine the need for increased privacy in particular circumstances:

…I guess it depends on the patient, too. Like, certain things could be private for one person and fine for another…

P10

A need for privacy was noted when consumers need to remove clothing:

…if they have to show me something on their body, for example, something discreet, might need to remove clothing … we'd come into this closed room…

P11

Assumptions were also made based on consumer age, experience and preferences:

…Our demographic is mostly elderly people, so we find in terms of privacy, they don't really mind. So they're not too fussed who we tell, or if we ring up the doctor; they're happy for us to do it … A lot of your younger people who are on antidepressants … tend to get a little bit more concerned if you ring up the doctors and things like that, but most of the elderly patients … rather you tell the doctor what they're all on and make sure they're ok than the younger people that we have…

P10

…Privacy is different for everyone; someone says ‘my diabetes or my heart problem is not private – you can tell them, you can discuss it,’ but others say ‘why did you tell someone I had a blood pressure problem?’ You know, privacy is different to different people…

P20

One pharmacist commented on strategies used to avoid opioid substitution therapy consumers encountering each other at the pharmacy:

…When the patient walks in, they've actually got a separate counter …. but there is … no room, so people can actually, on the other side, can still see what they are doing. So, the best thing is … for the pharmacist to look around, if it is someone that they know, we normally ask them first ‘Do you want me to do it now?’ and if then it's up to them whether they say ‘yay’ or ‘nay’. But it's normally only for the other [patient], because you know if they know each other, like they greet each other … and I normally tell the intern ‘just hold off until the other person that they know goes’…

P15

A number of pharmacists identified the management of unique privacy requirements of mental health consumers:

…We have a lot of mental health patients here…. they do need that one‐on‐one interaction more often than not…

P25

#### Consumers’ comments

In general, there was implied trust in pharmacists that consumers’ privacy would be protected. This was apparently based on staff professionalism, personal attributes and the personal experience of the consumer with the pharmacy:

…you trust them, that [pharmacy staff] don't go discussing you to other people. They're professionals, they shouldn't be doing that, and I don't think they would do that…

C24

…I trust [the pharmacist], especially the way he is not to reveal anything because that's the sort of person he is. I don't think he'd reveal anything. I have confidence in him, he's been very good…

C39

One consumer specifically identified that privacy is particularly important for consumers with sensitive health issues, such as mental health issues:

…I've got a bit of a problem with depression …. so that's why I come here. I feel quite at home here…

C54

Others indicated that if they felt uncomfortable, they would be proactive in asking for more privacy or would telephone the pharmacy. However, some consumers indicated they would not discuss personal issues to avoid being uncomfortable in the pharmacy environment and rather use the internet to obtain more information. One male participant (C36) moved away from the counter when staff had ‘women's talk’ with his wife.

Although some consumers had not given much thought to the subject matter, they felt assured staff would act appropriately:

…I guess it depends on what it's about. I remember speaking about my cholesterol tablets and I certainly didn't really mind about that, things like that. I guess if it's something more private, I'd probably prefer not to, but probably they wouldn't be asking in a big voice at the counter if it was something more private…

C26

## Discussion

This study combined the experiences and expectations of pharmacists and consumers regarding community pharmacy privacy practices; no other research to date has included this comparison of perspectives. Interviews with both parties provided valuable insights into facilitators and barriers in community pharmacy in achieving privacy. The use of private or semi‐private consultation areas for certain services and sensitive discussions was supported, although workforce issues should be considered for this model to be successful. Pharmacy staff adopted various practices to overcome privacy obstacles such as taking consumers to a quieter part of the pharmacy, lowering of voices or avoiding exposure of sensitive items through the use of packaging. These strategies demonstrate staff awareness of the need for privacy and the use of good communication and practice skills to enhance privacy. Pharmacists emphasized that professional judgement plays an important role in managing privacy in everyday practice considering the retail environment.

The consumers were, generally, comfortable with the level of privacy experienced in pharmacy practice, and there was implied trust in pharmacy staff to protect their privacy. However, examples were provided of privacy and confidentiality breaches that related to calling out consumers’ names to collect their medicines and mentioning the name of the medicine, overhearing conversations or visibility of products at the counter.

### Strategies to optimize pharmacy space to achieve privacy

Consumers generally received advice at the pharmacy counter. Some had experienced being moved aside to a quieter area, and this appeared universally to be initiated by the pharmacy staff member. The use of partitioned counselling booths or consultation rooms, although well accepted as a concept in pharmacies, was relatively new to consumers and highlighted the need for staff to further develop communication skills and workflow strategies. The existence of a separate consultation room in pharmacies appeared dependent on available floor space and recency of fit‐outs. Although the use of private consultation areas was positively perceived by most pharmacists, there were some reservations mainly related to the time a seated consultation would take and the cost for a second on‐duty pharmacist to continue with dispensing or other patient‐centred activities.^5^, ^6^ Of interest was that four of the pharmacies did not have private consultation areas as they did not provide in‐pharmacy medication review services. The possible reticence of a consumer to be singled out for consultation in a counselling booth was also raised, similar to mental health consumer research.^13^

Despite the prevalent use of the pharmacy counter for counselling, consumers appeared generally satisfied with this level of privacy and appeared comfortable, either considering their issue as not highly sensitive or refraining from discussing highly sensitive issues in the pharmacy. The level of comfort could also be a result of the clients being ‘regulars’ at those pharmacies, having rapport with the staff could improve their comfort and confidence with management of private issues. However, it is concerning to note that some consumers preferred not to discuss sensitive issues with a pharmacist due to perceived lack of privacy, and for these consumers, community pharmacies may not yet be perceived as a safe health space. This finding is similar to a study of public attitudes towards community pharmacies in Qatar, in which half of the participants stated that a lack of privacy was the most common barrier to asking pharmacists questions.^31^

Pharmacists highlighted the importance of privacy when dealing with special patient groups, and if computer screens are readable from where consumers stand. Pharmacy staff used various strategies to make visible information less obvious, such as asking consumers to sign for prescription medicine receipt in a more private area, or placing the medicines in bags before issue.

One unanticipated finding was the strategies employed by consumers in the event of needing to approach pharmacy staff about a private matter. These included telephoning rather than visiting the pharmacy and timing their visits to off‐peak periods to minimize witnesses to their conversation. These approaches were also utilized by pharmacists. Awareness of these consumer strategies by pharmacy staff can guide their service development. For example, if the pharmacy cannot physically accommodate a private counselling area, encouraging consumers to telephone, and ensuring telephone conversations are conducted in a quieter area of the pharmacy, could go some way towards meeting their needs for privacy.

Factors appearing to impact negatively on privacy in pharmacies included busy periods in the pharmacy, busy areas of the pharmacy (e.g. main medicines counter), and loud voices of staff, sometimes necessitated by hearing‐impaired consumers. Pharmacists could advertise the less busy periods as consultation times so consumers could return during those periods for greater privacy.

### Impact of practice changes on privacy requirements

It is apparent some consumers did not have an understanding of the changed role of pharmacists or pharmacists’ need for personal information to provide patient‐centred care. Some consumers did not perceive a need to provide sensitive information to pharmacy staff or simply would not share certain sensitive information with pharmacy staff if requested. These consumers commented that they would rather seek support or advice elsewhere, for example from doctors or the Internet. Indeed, inadequate privacy in pharmacies could impact on consumers’ lack of appreciation for the changed role of community pharmacists. Research with mental health consumers similarly identified a lack of knowledge and/or appreciation amongst some consumers and carers about advanced pharmacy services.^32^ A Welsh study investigating the importance of professionalism in pharmacy practice showed clients often lack trust in speaking with pharmacists if there is no privacy.^33^

Also evident in our data was differences between consumers in their expectations of privacy. For some, common conditions such as high blood pressure or high cholesterol required less privacy while other consumers expected any medical discussion to be conducted discreetly. Of interest was that some older consumers felt less need for discreet conversations compared to younger consumers, although other characteristics are also likely to be related to an individual's need for privacy.

Advanced services undertaken by pharmacists will bring to light new challenges relating to the management of privacy. Barriers to the disclosure of medical and personal information could impact on pharmacists’ ability to provide medication management services. This is particularly important as the profession is moving towards the provision of more professional services that involve talking to patients about not only their medical history but also lifestyle issues that could impact on their health care.

Recent Australian research indicated that consumers are often reluctant to engage with their pharmacist and ask questions about their health needs if they do not sense adequate privacy.^34^ Addressing perceptions about the role of community pharmacists is therefore especially important with the profession providing more disease state management services. American research found that lack of privacy not only impacts on consumers but could also impact on pharmacists’ confidence to counsel consumers, as community pharmacists were less confident in counselling clients with obesity if they perceived there was a lack of privacy.^35^

This study highlighted the need for pharmacy staff to apply professional judgement in the use of private areas and approaches to achieve privacy as well as the need to be sensitive to consumers’ preferences. Several practice‐related challenges were identified for consideration by pharmacy professional organizations. Specific communication skills to enhance privacy, such as lowering of voices, should be included in undergraduate training. There is also a need for on‐going research in pharmacy privacy as the profession moves towards increased provision of professional services.

### Limitations

This study involved a sample of metropolitan pharmacies. Due to logistical challenges, rural pharmacies were not included. While most of the consumers who participated in the interviews were regular clients of the pharmacy in which they were interviewed, privacy issues may become even more amplified in smaller communities. It is also possible that familiarity with the pharmacy and staff gave rise to positive reflections about the consumers’ comfort with sensitive discussions and trust in the staff. Pharmacists were involved in selection of consumers; this was in accordance with ethical approval for the study, such that the presence of a researcher in the pharmacy randomly approaching consumers would not create ill‐feeling amongst the clientele. To minimize pressure on consumers to ‘report on’ their pharmacy, consumers were asked about their experiences and values relating to privacy; some anecdotes relating to other people were also shared. Our data may not reflect other metropolitan centres in Australia, where different cultural mixes of clientele exist. In these cases, the importance of standards and staff training around management of privacy becomes even more paramount.

## Conclusion

Due acknowledgement and management of privacy can be challenging in the community pharmacy environment. On‐going work in this area is therefore important. As community pharmacy practice is increasingly involved in advanced medication management and disease state management services with unique privacy requirements, pharmacy layouts and systems to address privacy challenges must evolve. This requires a proactive approach in pharmacy design and the development of guidelines to rectify identified gaps in compliance. The information from this study provides valuable insight into community pharmacy experiences and expectations that should inform the profession in the development of privacy strategies.
